# Efficiencies of Super-Plasticizer on Rheology Properties of Fly Ash-Based Alkali-Activated Materials with Different Ms Waterglass Activators

**DOI:** 10.3390/polym15092054

**Published:** 2023-04-26

**Authors:** Dawang Zhang, Xuemei Sun

**Affiliations:** College of Materials Science and Engineering, Xi’an University of Architecture and Technology, Xi’an 710055, China

**Keywords:** alkali-activated fly ash fresh pastes, super-plasticizer, rheology properties, waterglass alkali activators

## Abstract

This study investigates the effects of five different super-plasticizers (SPs): melamine sulfonate (M), naphthalene-based (N), lignosulfonate (L), polyether-type (P-I), and polyester-type polycarboxylate super-plasticizers (P-II), on fly ash through fluidity, viscoelasticity, inter-microstructure, and mechanism of action (adsorption and zeta) experiments. Additionally, the stability of SPs on AAs was investigated in the ATR-FTIR experiment. The results show that most SPs were effective admixtures under high Ms (2.25) of waterglass (WG) alkali activators (AAs), while P-I SPs performed better under low Ms (1.0) of WG AAs in FA-AAM fly ash pastes. Meanwhile, the higher adsorption and zeta values of samples with P-I SPs were useful for the increase of mesh size of inter-particles and consequently promoted the rheology of FA-AAMs fresh pastes. The more stable structure (ether bond) and the formation of small functional groups (carboxylic acid groups) of P-I SPs in the AAs environment may be the main reasons for this.

## 1. Introduction

Alkali-activated materials (AAMs) consist of a polymeric Si-O-Al framework produced by geopolymerization between aluminosilicate precursors and activators; they have been considered to be sustainable and green alternatives to the high-carbon emissions of cement in urbanization construction materials, owing to their superior mechanical properties, durability, and fire, heat, acid, and corrosion resistance [1,2,3]. Differing from traditional cement materials, AAM fresh pastes exhibit significant disadvantages in their setting time, fluidity, and heat release, especially since their lower fluidity cannot meet the current pumping demand; this is one of the critical challenges which hinder their wider application. Despite this, based on previous investigations [4,5,6,7,8], adjustment of the physical and chemical properties of alkali activators (content, type, and chemical structure), as the main compositions determining the properties of AAM pastes, could improve the rheology properties of fresh pastes, such as by increasing NaOH AAs content, decreasing Ms (molar ratio between SiO_2_ and Na_2_O) of waterglass AAs, and using other lower-activity AAs. However, high-Na content in the NaOH AAs and low-Ms waterglass AAs easily leads to alkali-aggregate and carbonation reactions [9,10], and low-activity AAs (Na_2_CO_3_, Na_2_SO_4,_ and other AAs) could significantly reduce the early mechanical properties [11]. Thus, suitable adjustment methods of the rheology properties are essential in AAMs fresh pastes.

Water-reducing admixtures, also called super-plasticizers (SPs), are an important adjustment method and are used to improve the dispersion and workability of fresh cement pastes and to reduce the mixing water; this plays an important role in the preparation of high-performance cement-based cementitious materials. Unlike traditional OPCs, the environment provided by AAs, such as pore solution, pH, and ionic strength, poses a certain challenge to the effectiveness of super-plasticizers on the workability of AAM fresh pastes. Recently, SPs could significantly improve the workability of AAM fresh pastes activated by low-concentration NaOH AAs. For example, Palacios [12] found that Naphthalene (N) and Melamine(M) SPs could slightly increase fluidity (increases of up to 1%) in the slag-based AAMs with 4–5 wt.% NaOH AAs. Luukkonen [13] investigated the spread value of mortar with different SP types: Lignosulfonate (L), Sulfonated melamine-formaldehyde (SMF), Polycarboxylate esther (PCE), and Sulfonated naphthalene-formaldehyde (SNF) in the slag-based AAMs activated by 2.4 wt.% NaOH AAs and found that all SPs could improve the fluidity of mortar by up to 41%; the highest increases were observed with SNF and the L SPs. Ren [14], Pan [15], Yang [16], and Laskar [17] also found that N, PCEs, and other SPs are useful in improving the workability of FA-Slag-based AAMs fresh pastes activated by 3 wt.% NaOH AAs. Similar phenomena were found in investigations by Lei [18], Collins [19], Bakharev [20], and Li [21]. Based on the above investigations, most of the SPs could improve the workability of AAMs fresh pastes at low-NaOH AAs contents. In accordance with previous investigations [22,23,24,25], the solution condition provided by the activators determines the effectiveness of the super-plasticizer on the fluidity of fresh pastes, such as metal cations (Na^+^ and K^+^) and anionic groups (OH^−^, SiO_4_^2−^, CO_3_^2−^, SO_4_^2−^, and Al_2_O_3_^−^). Lower-concentrated anionic groups do not destroy the molecules of super-plasticizers, especially OH^−^ groups. However, for high-performance concrete, high- OH^−^ and activity aluminosilicate anionic groups are indispensable elements of AAMs for the enhancement of the geopolymerization process, especially for FA-based AAMs. Thus, the efficiencies of super-plasticizers in the high- OH^−^ and activity aluminosilicate anionic groups are of great importance for AAMs.

Waterglass AAs, which are complex mixed solutions containing types of polysilicon acid, alkali metal ions, and hydroxide ions, have become widely used AAs in high-performance AAMs. However, Douglas [26] and Palacios [12] found no effect of SPs in the AAM fresh pastes with waterglass AAs. The opposite phenomenon was found in the Yang [27] investigation. To date, however, research regarding the effect of super-plasticizers on AAMs only focused on a single structure of waterglass AAs and SPs. Thus, this study aims to investigate the effectiveness of five kinds of super-plasticizers on AAM fresh pastes with different Ms of waterglass AAs (Ms = 2.25, 2.0, 1.5, and 1.0). To accomplish this, tests measuring flowability, development of mesh size, and viscoelasticity of the fresh pastes were performed to study the effectiveness of super-plasticizers. Additionally, the interactions of AAM fresh pastes and super-plasticizer were surveyed via the stability of the super-plasticizer activator system, zeta potential, and absorption measurements.

## 2. Experimental

### 2.1. Materials

#### 2.1.1. Aluminosilicates Precursors

**Figure 1:**
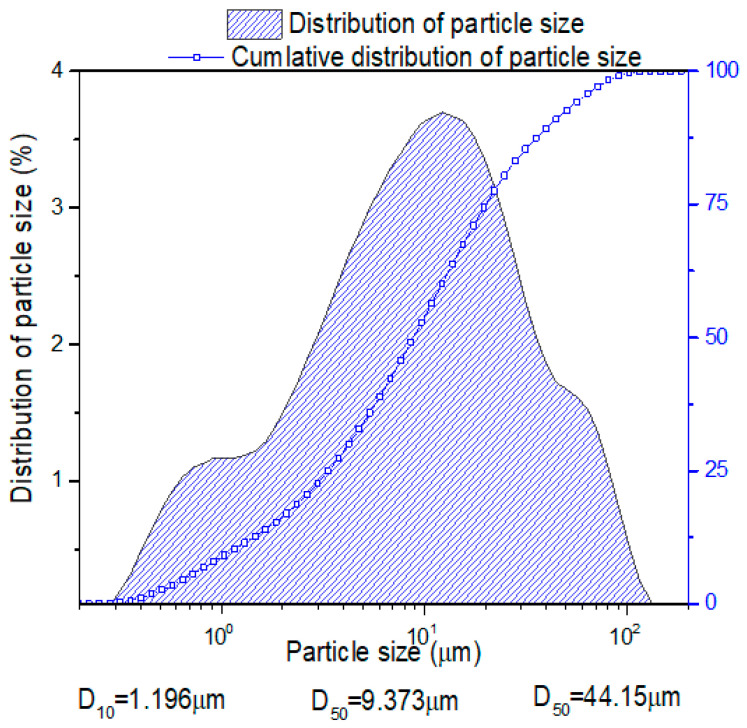
Cumulative distribution of particle size of fly ash materials.

Fly ash (FA, Sanmeng Xia company, He-nan, China) is the aluminosilicate precursor used as the binder material. Figure 1 shows the distribution of particle size of fly ash. The density and the average particle size D_50_ determined using laser diffraction of FA are 2.20 g·cm^−3^ and 9.73 μm, respectively. The chemical composition of FA is listed in Table 1.

#### 2.1.2. Alkali Activators

Waterglass (abbreviated as WG, Ms (molar ratio between SiO_2_ and Na_2_O) = 2.25, produced by Jiashan Yourui Refractory Co., Ltd., Beijing, China.) is the alkali activator (AAs). Moreover, four Ms of WG were adjusted by the sodium hydroxide, i.e., Ms = 2.25, 2.0, 1.5, and 1.0.

#### 2.1.3. Superplasticizers

Five kinds of SPs are selected to improve the flowability of FA-AAM fresh pastes in this study, such as melamine sulfonate (M), naphthalene-based (N), lignosulfonate (L), polyether-type (P-I), and polyester-type polycarboxylate super-plasticizers (P-II). Additionally, Table 2 shows the specifications of the super-plasticizers investigated in this study.

### 2.2. Mixing Methods of FA-AAM Fresh Pastes

Based on the mix design as shown in Table 3, firstly, the FA powder is added to the mixer; then, AAs are put into the mixer; after mixing, the SPs are finally added to the fresh pastes, which mix according to the procedure of GB/T8077-2012. Mixed fresh pastes would be used in the following measurements of rheological properties and other properties.

### 2.3. Testing Methods 

#### 2.3.1. Rheology Properties of FA-AAMs

##### Dispersing Effectiveness of SPs

In this study, the dispersing effectiveness of SPs on the FA-AAMs fresh pastes are evaluated by the fluidity via a mini-slump cone (height of 60 mm, upper diameter 36 mm, and bottom diameter of 60 mm). Following mixing, fresh pastes are put into the mixer.

##### Viscoelasticity Properties of FA-AAM Fresh Pastes with SPs

In this study, the viscoelasticity properties of FA-AAMs fresh pastes with SPs are poured into a 20 mL cylindrical glass bottle with a 25 mm diameter to be investigated through the Malvern Instruments (IEC60825) microrheology analyzer (Malvern, UK). Furthermore, the mesh size of the structure on the fresh pastes of FA-AAM is calculated by Equation (1) according to the G′ curves.

(1) $$\zeta =\sqrt[{3}]{\frac{dk_{b}T}{3\pi G^{\prime }}}$$

where $k_{b}$ is the Boltzmann’s constant, *T* is the absolute temperature, *d* is the dimensionality of the particle trajectories (usually 2 for microscopy), and ζ is the mesh size of the inter-structure of fresh pastes.

#### 2.3.2. Mechanism between SPs and FA-AAMs Fresh Pastes

##### Stability of SP on the AAs

The stability of SPs has been tested by composing mixed solutions of SPs and WG AAs of different structures (Ms = 2.25, 2.0, 1.5, and 1.0) in a 2:1 ratio. Apart from the physical stability (agglomeration, separation into a layer, and color change), the chemical stability (chemical structure) of SPs to study by the attenuated total reflectance Fourier transform infrared (ATR-FTIR) spectroscopy. 

##### Zeta Potential of FA-AAM Fresh Pastes

JS94H (Shanghai Zhongchen Digital Technology Equipment Co., Ltd., Shanghai, China) is used to measure the zeta potential of pastes. Meanwhile, in order to ensure the correctness of the experimental results, the test was repeated three times for each sample.

##### Adsorption of SPs on FA-AAM Fresh Pastes

In this study, the UV765-visible spectrophotometer (Shanghai Yidian Analytical Instruments Co., Ltd., Shanghai, China) was used to determine the adsorption of SPs on FA-AAM fresh slurry by the standard curve method, and the steps were as follows: the absorbance-concentration standard curve of SPs was plotted; then the sample to be measured was put into the corresponding standard curve to obtain the concentration of the sample; finally, the adsorption amount of fly ash surface water reducing agent was calculated.

## 3. Results and Discussing

### 3.1. Rheology Properties of FA-AAM with Different SPs

#### 3.1.1. Dispersing Effectiveness of SPs

**Figure 2:**
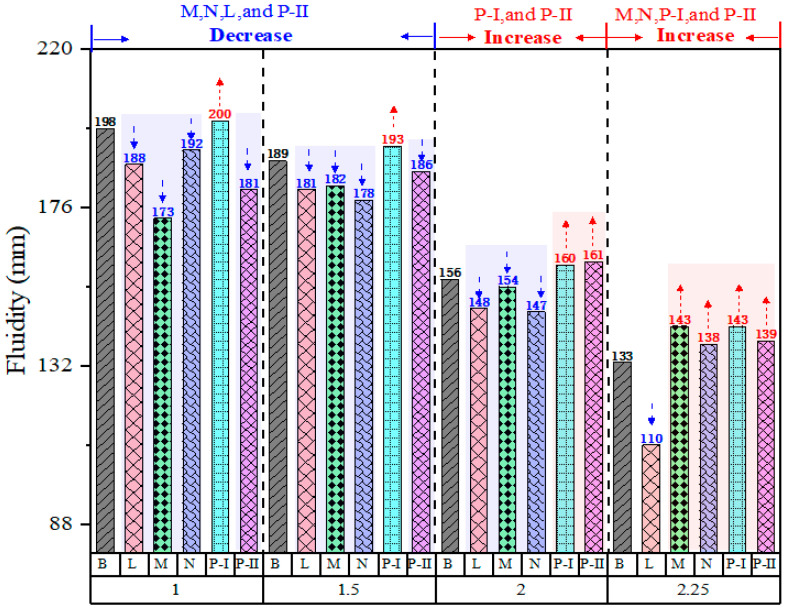
Effect of SPs on the fluidity of FA-AAM fresh pastes activated by the different Ms WG activators.

The effect of different SPs on the fluidity of FA-FA-AAMs with various Ms of WG is investigated to elevate the dispersing effectiveness of SPs in the fresh pastes, and the results are shown in Figure 2. In accordance with the results of fluidity experiments, the effectiveness of addition SPs gradually decreases with decreasing the Ms of WG. Under the Ms = 2.25 WG AAs, apart from the L SPs fresh pastes, the increase in the fluidity of FA-AAMs fresh pastes is 7%, 3%, 7%, and 6.7% when using M, N, P-I, and P-II SPs in reference to the control FA-AAMs fresh pastes without SPs, respectively. Additionally, the Ms of WG AAs has a relative significantly effect on the fluidity of FA-AAMs fresh pastes, i.e., with the decrease in the Ms of WG AAs, the effect of the SPs on the FA-AAMs fresh pastes gradually changes from increasing (Ms = 2.25 and 2.0) to decreasing (Ms = 1.5 and 1.0). Under the Ms = 1.5 of fresh pastes activated by WG AAs, the addition of L, M, N, and P-II SPs does not improve but decreases the fluidity of the FA-AAMs fresh pastes. As the Ms of WG AAs decreases, the negative effects of SPs become more pronounced in the fluidity of fresh pastes, and the percentage reduction is 5%, 12.6%, 3%, and 8.5% in the L, M, N, and P-II SPs comparing with the control FA-AAMs fresh pastes without SPs. However, as Figure 2 shows, the addition of P-I SPs significantly improves the fluidity of all fresh pastes. 

#### 3.1.2. Viscoelasticity of FA-AAM Fresh Pastes

**Figure 3:**
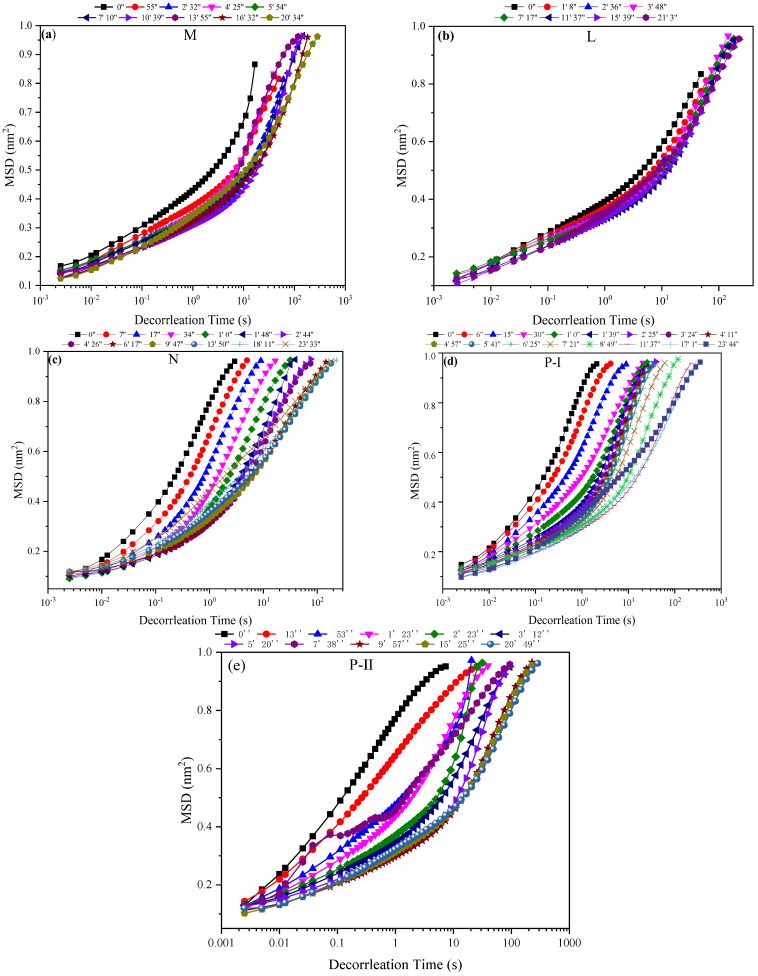
MSD curves of fresh pastes with different SPs in the Ms = 1.0 WG AAs: (**a**) M ; (**b**) L; (**c**) N; (**d**) P-I ; (**e**) P-II.

**Figure 4:**
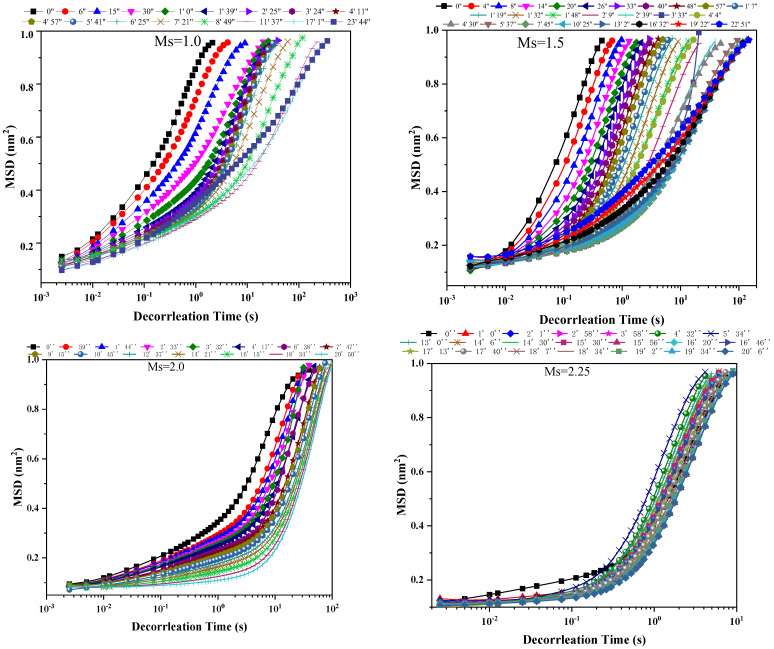
MSD curves of fresh pastes with P-I SPs in the different WG AAs.

**Figure 5:**
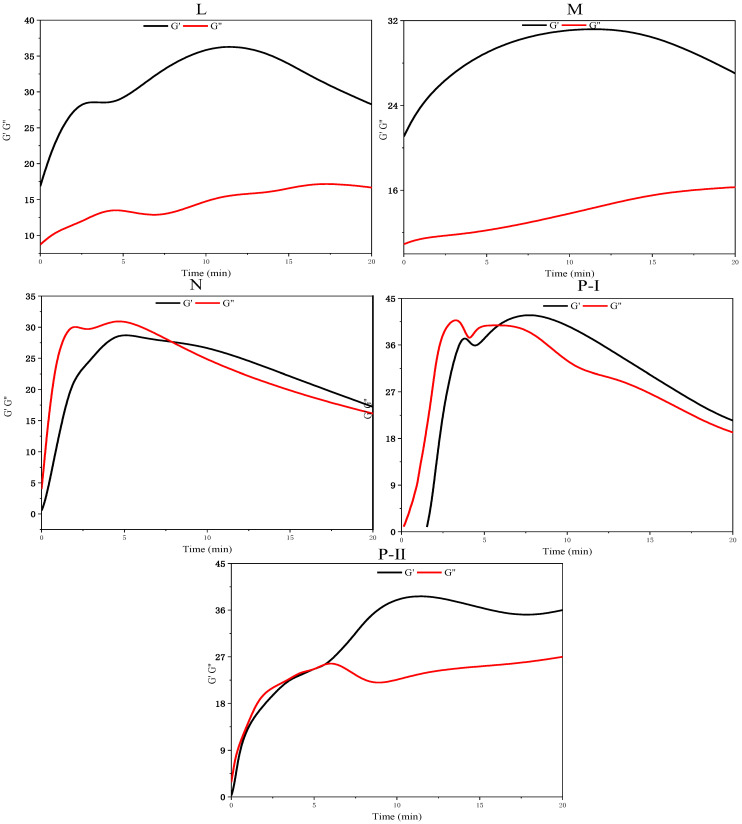
G′ and G″ of FA-AAMs fresh pastes with different SPs.

**Figure 6:**
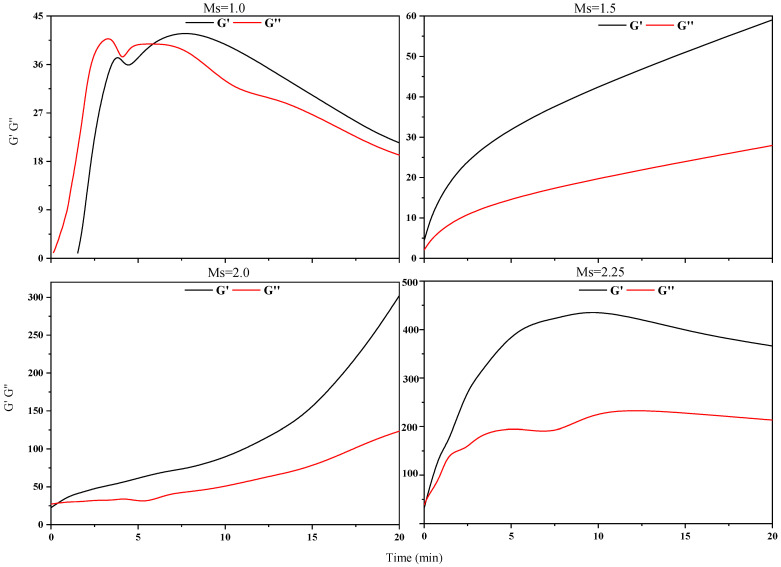
G″ and G′ curves of FA-AAM fresh pastes with P-I SPs under different Ms WG.

In this study, G′ (elastic modulus) and G″ (viscous modulus) over the range of frequencies during 20 min are used to better understand the viscoelasticity of FA-AAMs fresh pastes according to the MSD (diffusing wave spectroscopy, related to the particle motion) curves. Additionally, based on the experiment results of fluidity, although the low Ms of WG solutions are useful to improve the fluidity of fresh pastes, the effectiveness of SPs on the FA-AAMs fresh pastes significantly decreases, especially in the Ms = 1.0 WG AAs. However, the effectiveness of P-I SPs is less affected by the change of the Ms WG. Thus, in this part, the FA-AAM fresh pastes activated by Ms = 1.0 WG AAs with different SPs and the samples with P-I SPs activated by the different Ms WG are selected to investigate the viscoelasticity of FA-AAM fresh pastes. Furthermore, the experiment results are shown in Figure 3, Figure 4, Figure 5 and Figure 6. 

MSD curves of FA-AAMs fresh pastes are shown in Figure 2. Based on previous investigations [6,28], MSD curves, which are associated with particle motion, could be divided into three regions: (1) The movement of particles is free, the decorrelation time (*x*-axis) and MSD (*y*-axis) show a linear relationship in the curves. (2) Lower plateau was formed, attributed to the particles being blocked by the neighbor’s particle. (3) Liner region reappears after the long plateau period attributing to the formation of the “cage”. It is clearly seen that regions-I does not appear, but regions-II is directly presented in the MSD curves of FA-AAMs fresh pastes with L and M SPs, indicating the particles are blocked by the other particles to increase inter-particle friction leading to the decreases of fluidity. However, the MSD curves of samples with N, P-I, and P-II SPs exit a significantly liner region, consistent with the experiment results of fluidity.

Figure 4 shows the MSD curves of FA-AAMs with P-I SPs activated by the different Ms WG AAs. With the increases in the Ms of WG AAs, the MSD curves of fresh pastes gradually change from region I to regions II and III, suggesting the free particles are incrementally wrapped by other particles to form the “cage”. It results in a decrease in the fluidity of FA-AAMs fresh pastes with high Ms WG AAs.

In this study, G″ and G′ over a range of frequencies are used to understand the viscoelasticity of FA-AAM fresh pastes. Figure 5 and Figure 6 show the development of G″ and G′ of FA-AAM fresh pastes with different SPs and Ms of WG. All the G″ and G′ gradually increase with time and then reach a plateau indicating the development of microstructures. It is well known that G″ > G′ of samples in the early age, indicating the viscous behavior is pronounced in this stage. Oppositely, elastic behavior is the main behavior. As Figure 4 shows, apart from the samples with L and M SPs, the viscoelasticity of FA-AAMs fresh pastes with N, P-I, and P-II SPs present viscous behavior. This may associate with the effect of SPs on the fresh pastes, i.e., the steric hindrance effect could disperse the microstructure of pastes. In the case of samples with different SPs, compared with M and N samples, G′ curves start with a low value, which is in the range of 0–2. This phenomenon was consistent with the fluidity of pastes.

Additionally, the effect of Ms in WG on the viscoelasticity of FA-AAM fresh pastes is shown in Figure 6. As expected, high Ms of WG AAs results in the higher value of G″ and G′ of FA-AAM fresh pastes, in which the values of G″ and G′ could reach about 100. Moreover, the state of fresh pastes gradually changes from elastic to viscous behavior at the decrease of the Ms WG.

Based on the above investigations, the fluidity and viscoelasticity properties of FA-AAM fresh pastes are influenced by the inter-structure of particles forming the geopolymerization process at an early age. As is well known, solid particles of fresh pastes, which are dispersed in the AAs solution environment, could create new links between solid particles leading to the stringer “network structure” in the inter-structure. Thus, the size and information of the inter-structure will be investigated in this study. 

### 3.2. Size of Inter-Structure of FA-AAM Fresh Pastes

**Figure 7:**
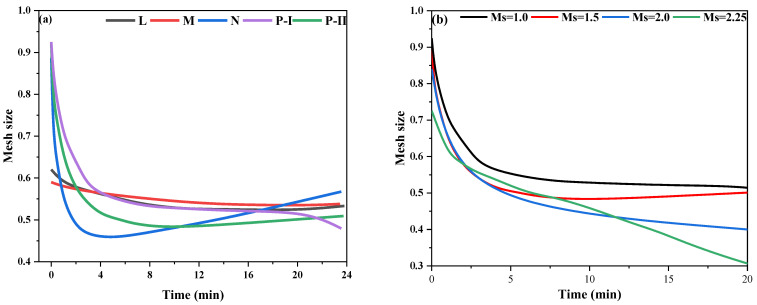
Mesh size of FA-AAM fresh pastes during the 20 min: (**a**) SPs; (**b**) Ms of WG AAs.

Similar to the cement-fresh pastes, solid particles were easily blocked by the neighbors to form the “large diameter particles” due to the physical and chemical force, also called the agglomerate [7,29,30]. For the microstructure of the fresh pastes, agglomerate directly affects the rheology properties by reducing the free water used for wetting and dispersing and hindering the movement of particles. Therefore, in this part, the mesh size is used to measure the inter-structure size of the agglomerate of FA-AAM fresh pastes according to previous investigations [28,31]. The result is shown in Figure 7.

As Figure 7a shows, the use of P-I, P-II, and N SPs was enough to increase the mesh size in the FA-AAM fresh pastes while maintaining an inter-structure higher than that of the control mix with L and M SPs. Additionally, the mesh size of fresh pastes gradually decreases with Ms of WG AAs, as shown in Figure 7b. This pattern is in accordance with the rheology of FA-AAM fresh pastes. Under the smaller mesh size of inter-structure in the FA-AAMs fresh pastes, solid particles are tightly gathered together to hinder the movement of particles and influence the rheology properties of fresh pastes. 

In conclusion, the larger mesh size of FA-AAMs fresh pastes has free space for solid particles leading to a liquid-like state of the suspension (viscous state, G″) and reduces the chance of collision between particles reducing the internal friction and improving the fluidity of fresh pastes. The particle–particle interaction forces influence the mesh size between structures, such as the mechanisms between SPs and solid particles are also influencing factors, especially the electrostatic repulsion and adsorption of SPs. Thus, the mechanism between SPs and fresh pastes is shown in Section 3.3.

### 3.3. Mechanism between SPs and Fresh Pastes

**Figure 8:**
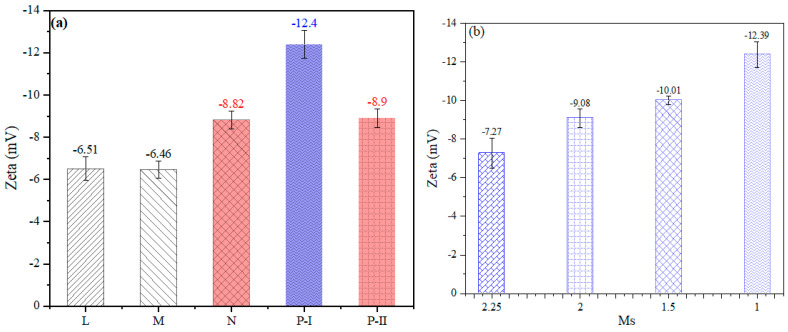
(**a**) Efficiency of different SPs on the zeta potential of FA-AAM fresh pastes; (**b**) efficiency of different molarity on the zeta potential of FA-AAM fresh pastes with P-I.

Figure 8 shows the zeta potential of fresh pastes in different SPs. A higher absolute value of zeta is obtained in the samples with P-I SPs compared with other SPs, which could reach −12.4 mV. This phenomenon relates to the electrostatic repulsion provided by the ions of AAs solutions, and SP absorbed on the particles. Thus, the adsorption of SPs on the particles is the main reason for the electrostatic repulsion of fresh pastes. According to the results of absorption (Table 4), the highest value 0.1682 g/g is obtained in the P-I samples, resulting in the increasing of electrostatic repulsion to increase the mesh size of inter-structure to reduce the internal friction of fresh pastes, which explains why such P-I SPs work efficiently in FA-AAM pastes. A similar phenomenon is found in the FA-AAMs with P-I SPs activated by the different Ms WG AAs. However, compared with conventional cement fresh pastes, although the SPs in FA-AAMs fresh pastes have a higher adsorption value, it does not significantly improve the rheological properties, i.e., fluidity, viscoelasticity, and microstructure. 

It is well established that the physical and chemical stability of SPs controls the stability and hence the dispersing performance of SPs [32]. Thus, it may be the main reason for the above phenomenon. For this reason, the stability of SPs in the alkali activators is performed with the aim of revealing the physical and chemical change in the structure of SPs in high environments, and the results are shown in Section 3.4.

### 3.4. Stability of SPs

**Figure 9:**
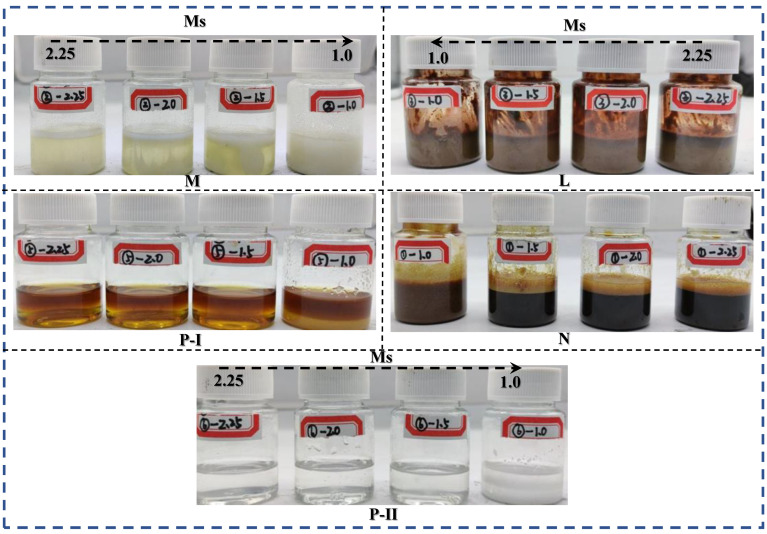
Appearance of SPs in different AAs environments.

**Figure 10:**
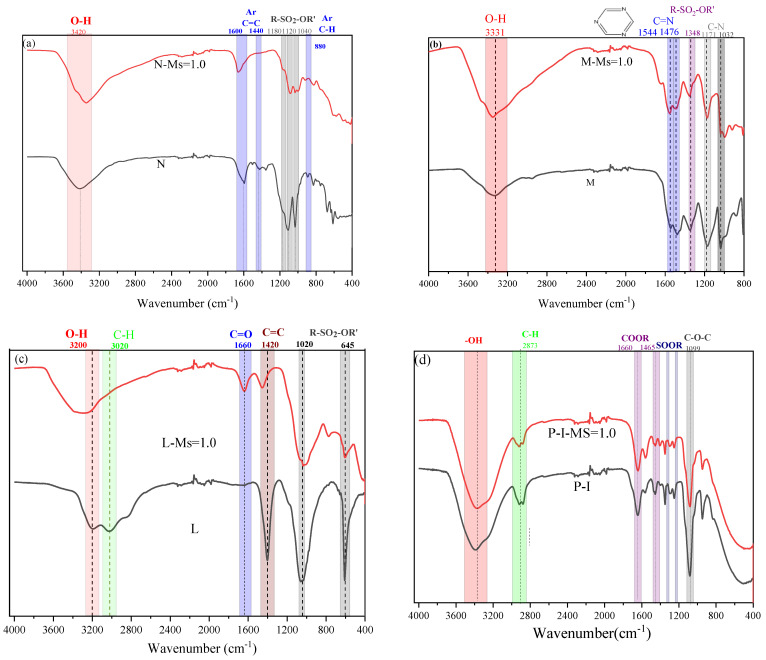

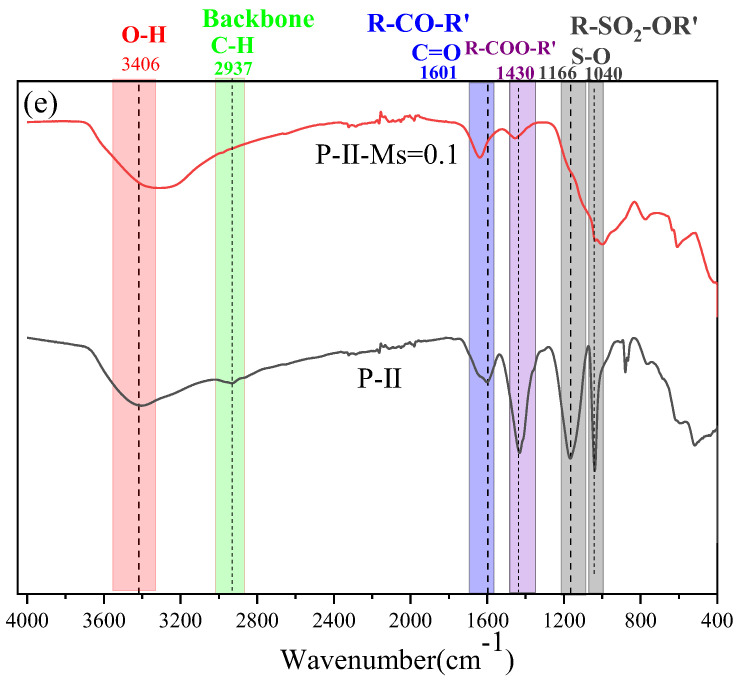
ATR-FTIR spectra of SPs in AAs environment.

In this part, the chemical and physical properties of SPs in the AAs environment are used to evaluate the stability of SP. Those experiment results are shown in Figure 8 and Figure 9. Noticeable color, agglomeration, and separation changes are found in the physical properties of SPs in the AAs environments (Figure 9). Under higher Ms WG AAs (Ms = 2.25), there is a slight physical change in the SPs systems. Nevertheless, with the decreasing of Ms, clear agglomeration gradually formed in the samples. Until reaching the Ms = 1.0, the separation into layers phenomenon is found in the samples. Among the five SPs, M, and N SPs exit significant color change, such as the color of M SPs gradually changing from colorless to yellow and N SPs turning from black to red. Additionally, other SPs exit significant phenomena of agglomeration and separation in the lower Ms. AAs solutions. From this ensemble of results, the molarity of Ms = 1.0 is selected for further investigations as it represents the turning point of the physical properties influencing the properties of fresh pastes. Thus, the chemical stability of SP with Ms = 1.0 of WG, which was investigated by ATR-FTIR, is shown in Figure 10. 

As Figure 10 shows, the significant transformation of SPs focuses on the main functional peak of the group after 24 h interaction with activators (Table 5): 

(1) For the N SPs (Figure 10a), Ar-SO_2_-O^−^M^+^ and Ar groups exit obviously changes: two main characteristic peaks (1600 and 1440 cm^−1^) of Ar groups gradually turn to the 1600 cm^−1^, which indicates the disappearance of the conjugated group on the Ar. Furthermore, the intensity of the Ar-SO_2_-O^−^M^+^ characteristic peak (1180 cm^−1^, 1120 cm^−1^, and 1040 cm^−1^) is significantly reduced. The above changes in the characteristic peak indicate that the SO_2_-O^−^M^+^ on the Ar is stripped and decomposed into sulfonate; 

(2) For the M SPs, the peak intensity and width of the C-N group significantly decrease after the As environment, i.e., –C-N-C-S, -C-N-C, and –C-N-C-O^+^. This phenomenon may be related to the breakage of the “=C-N-C-S-Na” absorption group leading to the lower absorption amount on the surface of particles; 

(3) For the L SPs, the intensity of characteristic peaks of R-SO_2_-OR′ and C=C of Ar bond significantly decreases under the WG AAs solutions. It indicates the structure of the R-SO_2_-OR′ and C=C bond was destroyed to form a larger number of small molecule structures under the high alkali environment;

(4) For polyether-type (P-I) and polyester-type polycarboxylate super-plasticizers (P-II), the R-COO-R’ and R-SO_2_-O-R of P-II, acting as an adsorption group, disappear in the WG AAs solution after 24 h. It results in the decreasing effectiveness of P-II SPs in the FA-AAMs. However, the main characteristic peak- C-O-C group and R-COO-R’ group of P-I slight change, leading to a significant decrease in the effect of SPs on the rheological properties of fresh pastes.

## 4. Conclusions

In this study, the efficiencies of super-plasticizer (melamine sulfonate (M), naphthalene-based (N), lignosulfonate (L), polyether-type (P-I), and polyester-type polycarboxylate super-plasticizers (P-II)) on rheology properties of the fly ash-based alkali-activated materials with different Ms waterglass activators was investigated. The study resulted in the drawing of the following conclusions: 

(1) Efficiencies of SPs: based on the fluidity experiments, when the Ms reach 1.0, only the P-I SPs could slightly improve the fluidity of FA-AAMs fresh pastes;

(2) Compared with the other SPs, P-I SPs absorbing on the surface of solid particles improve the absolute value of zeta potential leading to an increase in mesh size of inter-particles and consequently promoting the rheology of FA-AAMs fresh pastes;

(3) Under the FA-AAMs fresh pastes with Ms = 1.0 WG AAs, the main characteristic R-COO-R’ group of polyether-type SPs (P-I) has a slight change, which may be the main reason for the significant decrease in the effect of SPs on the rheological properties of fresh pastes.

## Figures and Tables

**Table 1:** Chemical composition of raw materials/wt%.

	CaO	SiO_2_	Al_2_O_3_	MgO	Fe_2_O_3_	SO_3_	Na_2_O	K_2_O	LOI
FA	4.23	49.76	24.58	0.857	1.82	2.25	0.317	4.607	11.57

**Table 2:** Physical properties of super-plasticizers.

SP	Chemical Base	Color	Commercial Name	Company
M	Melamine sulfonate	White	SM	Jinan Qingtian Chemical Technology Co., Ltd., Beijing, China
N	Naphthalene sulfonate	Light brown	FDN	Shanghai Yunzhe New Material Technology Co., Ltd., Shanghai, China
L	Sodium lignin sulfonate	Dark brown	CMN	Shangdong Yueqi Chemical Co., Ltd., Shandong, China
P-I	Polyether-type SP	Brown	PC-52	Tangshan Longyi Technology Development Company, Tangshan, China
P-II	Polyester-type SP	White	PC-14	Tangshan Longyi Technology Development Company, Tangshan, China

**Table 3:** Mix design of FA-AAM fresh pastes/g.

SP	FA	Water	SP/FA/%	WG AAs				Mix
				2.25	2.0	1.5	1.0	
M	100	35	0.8	12				M-2.25
					12			M-2.0
						12		M-1.5
							12	M-1.0
N	100	35	0.8	12				N-2.25
					12			N-2.0
						12		N-1.5
							12	N-1.0
L	100	35	0.3	12				L-2.25
					12			L-2.0
						12		L-1.5
							12	L-1.0
P-I	100	35	0.3	12				P-I-2.25
					12			P-I-2.0
						12		P-I-1.5
							12	P-I-1.0
P-II	100	35	0.3	12				P-II-2.25
					12			P-II-2.0
						12		P-II-1.5
							12	P-II-1.0

**Table 4:** Adsorption of SPs on the FA-AAM fresh pastes.

Mix	C_0_/(g/L)	C/(g/L)	q/(g/g)
L-1.0	0.2	0.0556	0.1444
M-1.0		0.0578	0.1422
N-1.0		0.0528	0.1472
P-I-1.0		0.0318	0.1682
P-I-1.5		0.0354	0.1646
P-I-2.0		0.0398	0.1602
P-I-2.25		0.0484	0.1516
P-II-1.0		0.0524	0.1476

Co is the concentration of SPs. C is the concentration of fresh pastes. q is the adsorbed amount of SPs on the solid wastes.

**Table 5:** Main characteristic peak of SPs [12,33,34,35,36].

Group	Positions of Peak
OH	3420 cm^−1^
-C=C of Ar,	1600 and 1440 cm^−1^
Ar-SO_2_-O^−^M^+^	1356, 1180, and 1120 cm^−1^
–C-H of Ar	880 cm^−1^
C-H	3020 cm^−1^,
CH_3_-O	1420 cm^−1^
R-SO_2_-OR^’^	645 cm^−1^, 1020 cm^−1^, and 1348 cm^−1^
C=N bonds	1544 and 1476 cm^−1^
C-N bond	1171 and 1032 cm^−1^
C=O of the carboxylic	1727 cm^−1^
*vas* and vs. of COOR	1660 and 1465 cm^−1^
